# From Risk-Sensitive Foraging to Scalable Portfolio Rebalancing: State-Conditioned Artificial Bee Colony Optimization of Cost-Aware Omega

**DOI:** 10.3390/biomimetics11100724

**Published:** 2026-10-10

**Authors:** Haoran Nie, Yuanbo Lu, Hua Xie, You Wu

**Affiliations:** 1 School of Banking and Finance, Business School, University of New South Wales, Sydney, NSW 2052, Australia; 2 College of Automotive and Energy Engineering, Tongji University, Shanghai 201804, China; 3 Public Infrastructure Department, Ganzhou Polytechnic, Ganzhou 341000, China; 4 Software College, Northeastern University, Shenyang 110169, China

**Keywords:** artificial bee colony, risk-sensitive foraging, biomimetic optimization, Omega ratio, portfolio rebalancing, cardinality constraint, transaction costs, adaptive search

## Abstract

This study examines whether candidate-level gain–loss information improves search for cost-aware portfolio rebalancing. We propose Risk-Sensitive Foraging Artificial Bee Colony (RSF-ABC) for long-only portfolios with exact cardinality and active-weight bounds. A bounded state derived from the Omega objective controls the probability of heavy-tailed exploration versus conservative best-guided search. A state-independent mixture of the same operators isolates the value of state conditioning. Monthly evaluation on U.S. industry portfolios uses 504-day formation windows and proportional transaction costs. Mean net Omega is 1.1635 for RSF-ABC, 1.1284 for Standard ABC, 1.1342 for Fixed-Mix-ABC, 1.1410 for particle swarm optimization, and 1.1450 for differential evolution. Monthly turnover is 0.4780 versus 0.5570 for Standard ABC. The proposed method leads in five of seven boundary conditions; deterministic alternatives lead in two. At 49 assets, its reported median runtime is 1.11 s versus 1.08 s for Standard ABC. These results support the evaluated state–operator coupling.

## 1. Introduction

Portfolio rebalancing requires an investor to choose which assets to hold, how much capital to allocate, and how far to move from the existing portfolio. An allocation that appears attractive before trading can become less useful after transaction costs, while cardinality and weight bounds restrict the set of admissible decisions. This study addresses these coupled decisions by maximizing the empirical Omega ratio after a turnover-based cost adjustment. The objective is to determine whether candidate-level gain–loss information can improve the allocation of search effort in an Artificial Bee Colony (ABC) optimizer.

Risk-sensitive foraging motivates the proposed control rule. Recent experiments relate honey-bee risk responses to seasonal resource scarcity [1], while the critical review by Houston and Rosenström [2] explains why a single energy-budget rule cannot account for all observed behavior. We transfer a specific functional relation: an internal state changes the probability of selecting a variable action. The financial gain–loss state used here is an engineering representation of that relation, rather than a measurement of biological energy.

Canonical ABC alternates employed-bee, onlooker-bee, and scout-bee phases [3]. Its exploration–exploitation balance has motivated substantial methodological development [4]. Wang et al. [5] examined labor division, while Yang et al. [6] adapted the choice between one-dimensional and all-dimensional search to variable correlations. Recent work studies switching based on problem features [7], and NeurELA learns landscape representations for optimizer configuration [8]. Adaptive search control is therefore an established research direction. The question here is whether a candidate’s objective-derived gain–loss state provides useful feedback for selecting its next move when the available operators are held fixed.

The portfolio literature provides the problem setting and algorithmic precedents. Erwin and Engelbrecht [9] reviewed metaheuristics for portfolio optimization. Kalayci et al. [10] combined feasibility enforcement with infeasibility toleration, Cura [11] developed an ABC method for cardinality-constrained portfolios, and Wei et al. [12] investigated neighborhood search. Hybrid metaheuristics [13], Mayfly search [14], and a transaction-cost-aware memetic method [15] offer alternative approaches to joint asset selection and weight allocation. Together, these studies make constraint handling a central part of the comparison: a change in repair, objective, or search budget could otherwise be mistaken for a benefit from state conditioning.

Recent portfolio research connects allocation quality to the information and decision model used. Deep parametric portfolio policies learn nonlinear mappings from firm characteristics to weights [16]. Robust reinforcement learning addresses market-impact uncertainty [17], and Bayesian optimization exploits the evaluation structure of risk-constrained allocation [18]. Online portfolio selection with machine-learning predictions studies how forecast quality affects performance guarantees [19]. AlphaGAT separates factor extraction from adaptive allocation [20], while heuristic-guided inverse reinforcement learning incorporates domain knowledge into the reward and policy [21]. These approaches address different sources of decision quality. Our contribution concerns feedback within the optimizer: the current candidate’s gains and losses regulate search under a fixed portfolio objective. Their published performance values cannot be compared directly across the differing objectives, costs, data, and information sets.

The Omega ratio compares expected gains above a chosen threshold with expected shortfalls below it [22]. It provides both the portfolio objective and the quantities used to construct the gain–loss state. Compatible continuous Omega problems admit linear or related reformulations [23,24], but the interpretation of an optimum and its sensitivity to the return distribution and threshold require care [25,26,27]. Our setting retains long-only positions, exact cardinality, active-weight bounds, and trading costs. Simple diversification [28] and a mixed-integer Omega comparator test whether a stochastic search improvement also matters relative to financial alternatives.

RSF-ABC normalizes the candidate’s gain–loss balance to a bounded state and maps that state through a sigmoid to an exploration probability. The exploratory branch uses a clipped Student-t multiplier; the conservative branch moves toward the current best source. Both branches share the same feasibility repair and acceptance rule. A matched Fixed-Mix-ABC control uses the same branches with a constant probability. This comparison isolates state dependence from a change in branch composition, while additional ablations examine heavy-tailed exploration and switching separately.

The study asks whether objective-derived feedback improves a fixed pair of search operators, how that improvement compares with alternative optimizers, and what computational cost it entails. Its contributions are as follows.

1. A specified state–operator coupling: The candidate’s normalized Omega gain–loss balance controls the probability of two neighborhood moves. The matched-mixture control holds the moves fixed, making the incremental role of state dependence testable.
2. An evaluation across algorithm families: Particle swarm optimization and differential evolution share the stochastic evaluation budget, alongside four ABC controls and equal-weight, minimum-variance, and mixed-integer Omega portfolios. These comparisons assess objective quality; the available runtime results are considered separately.
3. A portfolio study with explicit operating conditions: Monthly walk-forward evaluation includes transaction costs, drifted pre-trade weights, exact feasibility, and seven boundary conditions. A fixed-budget scaling diagnostic examines larger arrays. The results identify a quality–cost trade-off within these conditions, rather than a general efficiency advantage.

## 2. Materials and Methods

### 2.1. Problem Definition and Assumptions

At the end of each formation window, an investor observes historical asset returns and the portfolio held immediately before trading. The decision is a new vector of portfolio weights, including the choice of exactly *K* assets from a universe of *N*. The goal is to maximize the formation-window Omega ratio after accounting for the turnover required to reach that target. The target is then evaluated over the next calendar month, whose returns are unavailable when the decision is made. Repeating this procedure produces a sequence of linked rebalancing problems because each decision changes the portfolio carried into the next month.

The model assumes long-only positions, full investment after rebalancing, and fixed lower and upper bounds on every selected weight. Feasibility requires 1 ≤ *K* ≤ *N* and *K*ℓ ≤ 1 ≤ *Ku*. Fractional allocations are allowed; minimum lot sizes, leverage, and short selling are excluded. The investor is treated as a price taker, and the one-way transaction-cost rate is constant within each experimental condition. The cost model excludes market impact, bid–ask variation, taxes, and capacity limits.

Historical daily observations receive equal weight in the empirical objective; normality of returns is not required. All estimated quantities use the current formation window. The initial portfolio is cash, and subsequent decisions use drifted pre-trade weights. Rebalancing occurs monthly. The formation objective spreads the anticipated trading charge over 21 days, whereas the holding-period accounting charges it once at the start of the month. This distinction is a modeling approximation, not an assumption that future returns or the future month length are known.

### 2.2. Mathematical Model

Let ***r****_t_* ∈ ℝ*^N^* denote the vector of daily asset returns in a formation window, and let ***w*** ∈ ℝ*^N^* denote the target portfolio. Portfolios are long-only, fully invested, and exactly cardinality constrained:

(1) $$W=\{w:\sum_{j=1}^{N}w_{j}=1,\;\sum_{j=1}^{N}z_{j}=K,\;lz_{j}\leq w_{j}\leq uz_{j},\;z_{j}\in \{0,1\}\}.$$

The main condition uses *N* = 49 industries, *K* = 10 active positions, lower bound ℓ = 0.02, and upper bound *u* = 0.30. These restrictions create a concentrated industry portfolio while excluding negligible selected positions and single-industry dominance. All stochastic methods use the same feasibility operator: it retains the *K* largest coordinates of a raw candidate and projects them onto the bounded simplex. Holding this repair fixed prevents constraint handling from becoming a hidden source of performance differences. Explicit feasibility procedures are consequential in ABC portfolio optimization [10], and cardinality combined with trading costs creates a substantive mixed search problem [15].

At each rebalance, ***w***^−^ denotes the risky-asset weights immediately before trading and *a*^−^ denotes the pre-trade cash weight. One-way turnover is

(2) $$q(w,w^{-},a^{-})=\frac{1}{2}({∥w-w^{-}∥}_{1}+|a^{-}|).$$

The first rebalance begins from cash, so ***w***^−^ = 0 and *a*^−^ = 1. Later pre-trade weights are obtained by drifting the previous target portfolio through the realized holding-month asset returns. A proportional one-way cost *c* is charged at each rebalance. The primary value is 25 basis points.

For a formation window with *T* daily scenarios, daily threshold *τ*, and assumed holding-month length *H* = 21, the cost-adjusted excess return of candidate ***w*** in scenario *t* is

(3) $$x_{t}(w)=r_{t}^{⊤}w-\frac{c\;q(w,w^{-},a^{-})}{H}-\tau .$$

The formation objective amortizes the one-time rebalance cost across 21 daily scenarios. This proxy exposes turnover to the optimizer without using the realized length or returns of the future holding month. It should not be interpreted as realized implementation shortfall: the out-of-sample path charges the proportional cost once on the first holding day, and the model omits bid–ask variation, market impact, taxes, and capacity. The fixed value *H* = 21 is a design convention for a typical trading month, not an estimate fitted to future observations.

Mean gain and loss relative to the threshold are

(4) $$G(w)=\frac{1}{T}\sum_{t=1}^{T}[x_{t}(w){]}_{+},\;\;L(w)=\frac{1}{T}\sum_{t=1}^{T}[-x_{t}(w){]}_{+}.$$

where [*y*]_+_ = max(*y*,0). The empirical objective is

(5) $$\Omega (w;\tau )=\frac{G(w)}{L(w)+\varepsilon }.$$

with *ε* = 10^−12^. The primary threshold is zero daily return. One boundary condition instead uses the current formation-window mean return of the equal-weight portfolio, so the alternative threshold is also estimated without look-ahead.

The monthly optimization problem is therefore

(6) $$w^{⋆}\in \underset{w\in W}{\arg\;\max}\Omega (w;\tau ).$$

### 2.3. Basic Artificial Bee Colony Algorithm

ABC is selected because its population and local neighborhood updates combine directly with portfolio repair, while the employed, onlooker, and scout phases provide a framework in which one control component can be changed [3]. Constrained-portfolio applications provide relevant precedents [10,11,12]. Particle swarm optimization, differential evolution, and financial comparators are retained to test the resulting performance beyond the ABC family.

In the basic ABC neighborhood, an employed bee selects a different source *k* and a coordinate *j*, then generates a trial vector that differs from source *i* at that coordinate:

(7) $$w_{\mathrm{ij}}^{\prime }=w_{\mathrm{ij}}+\phi (w_{\mathrm{ij}}-w_{\mathrm{kj}}),\;\;\phi ∼U(-1,1).$$

The trial replaces the source if it improves the objective. Onlookers allocate additional trials to promising sources, and scouts replace sources whose unsuccessful-trial counters reach a limit. In the portfolio implementation, raw trials are repaired before evaluation, onlookers use descending objective ranks, and lower turnover breaks numerical objective ties. These common implementation choices are retained across the ABC variants. For a source with rank *r_i_* (rank 1 is best), the onlooker selection probability is $(P-r_{i}+1)/\sum_{h=1}^{P}h$.

### 2.4. Risk-Sensitive Foraging Artificial Bee Colony

The proposed state uses the same gain and loss terms as the empirical Omega objective:

(8) $$B(w;\tau )=\frac{G(w)-L(w)}{G(w)+L(w)+\varepsilon }.$$

The state approaches −1 when threshold shortfalls dominate and +1 when gains dominate. Unlike Omega, the normalized difference is bounded and symmetric around equal gain and loss. It is not a biological measurement. It translates the functional idea of a resource budget into an application-native variable that can condition search.

For source ***w****_i_*, partner ***w****_k_*, current best ***w***^⋆^, and coordinate *j*, RSF-ABC changes the neighborhood operator while retaining Algorithm 1. Its exploration probability is

(9) $$p_{\mathrm{explore}}(w_{i})=\frac{1}{1+\exp\{\kappa B(w_{i};\tau )\}}.$$

The primary algorithm uses κ = 4. Values of κ = 2 and κ = 8 define shallower and steeper diagnostic sensitivities. A negative gain–loss state raises exploration probability, a positive state favors conservative search, and a balanced state gives equal branch probabilities. The sigmoid is an engineering choice for a smooth bounded response; the foraging literature motivates state dependence without prescribing this functional form [2].
**Algorithm 1.** Standard ABC used in the portfolio comparisonInput the formation returns, pre-trade portfolio, constraints, population size *P*, evaluation budget *F*, and scout limit.Initialize and evaluate a common feasible population; set evaluation count to *P* and unsuccessful-trial counters to zero.While evaluations remain, perform the employed, onlooker, and scout phases below.Employed phase: For each source, select a different partner and one coordinate; generate a basic ABC trial.Repair the trial to exact cardinality and the bounded simplex; evaluate cost-aware Omega and turnover; increment the evaluation count.Accept an improving trial, or a tied trial with lower turnover; reset its counter on acceptance and increment the counter otherwise. Update the best-so-far source when improved.Onlooker phase: compute descending-rank probabilities and make up to *P* sampled-source trials using steps 4–6.Scout phase: Replace each source reaching the unsuccessful-trial limit with a newly sampled feasible source, evaluate it, reset its counter, and update the best-so-far source if improved.Check the remaining budget before every trial or scout evaluation, including an incomplete final phase.Return the best feasible portfolio found within *F* objective evaluations.

When exploration is selected, RSF-ABC applies a heavy-tailed coordinate move:

(10) $$w_{\mathrm{ij}}^{\prime }=w_{\mathrm{ij}}+\phi (w_{\mathrm{ij}}-w_{\mathrm{kj}}),\;\;\phi ∼\mathrm{clip}(t_{3},-4,4).$$

The heavy-tailed multiplier permits occasional steps beyond the range of the canonical uniform perturbation, while clipping limits numerical extremes. The conservative branch moves the selected coordinate toward the current best source:

(11) $$w_{\mathrm{ij}}^{\prime }=w_{\mathrm{ij}}+\rho (w_{j}^{⋆}-w_{\mathrm{ij}}),\;\;\rho ∼U(0,0.5).$$

**Figure 1:**
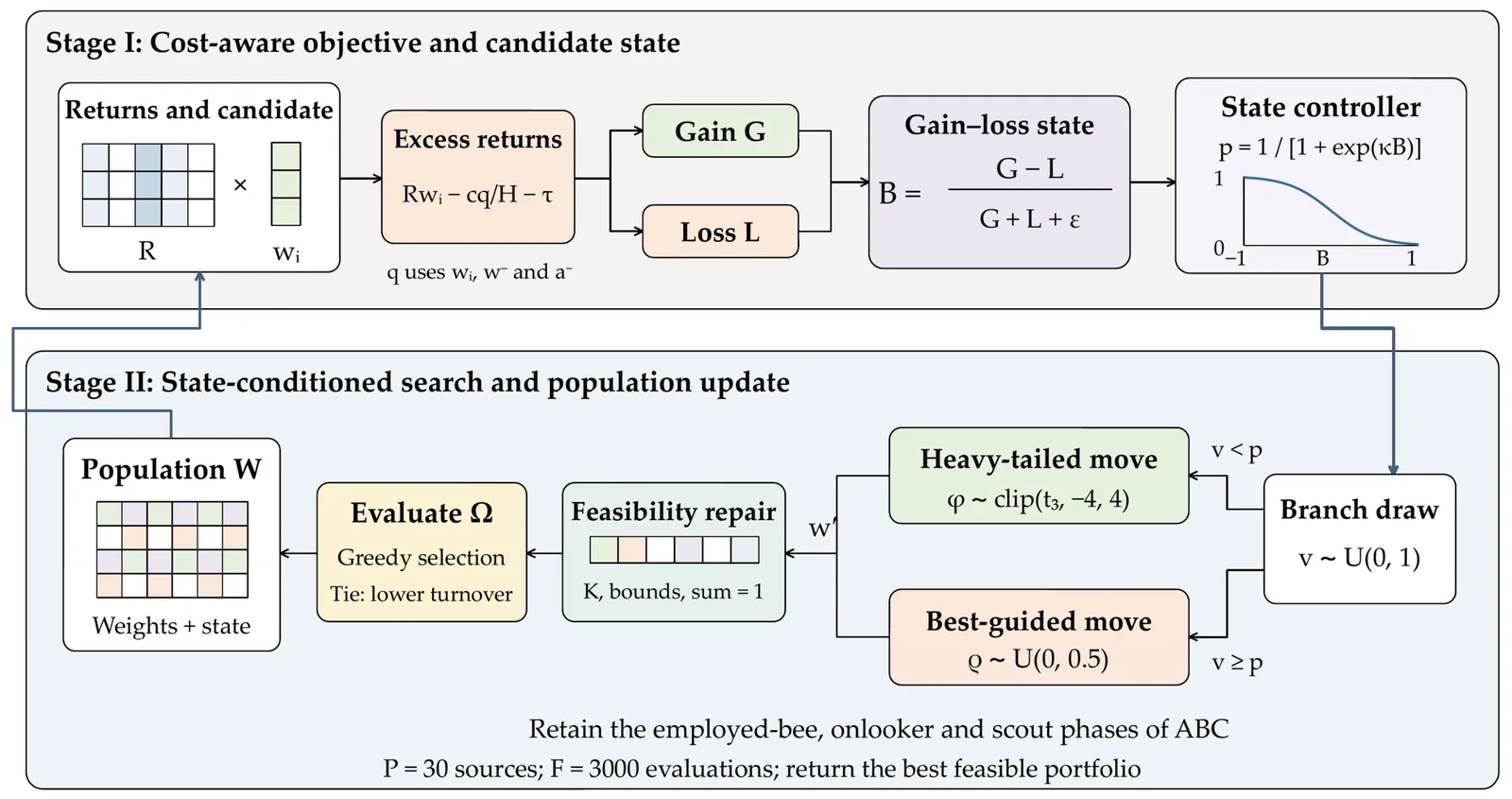
State-conditioned search. Stage I calculates cost-adjusted excess returns, gains, losses, and the candidate’s state; Stage II uses that state to choose a search branch before feasibility repair and greedy selection. Black arrows indicate computation within stages; blue arrows carry the state-dependent decision forward and return updated candidates. Green boxes denote gains and exploration; peach denotes excess returns, losses, and best-guided search; purple denotes the state and controller; pale green denotes repair; and yellow denotes evaluation. Gray and pale-blue backgrounds separate the stages.

Every raw candidate is repaired to 𝒲 before evaluation. Greedy replacement compares empirical Omega and then prefers lower turnover when objective values differ by no more than 10^−12^. The population size is 30, the unsuccessful-trial limit is 100, and each run uses exactly 3000 objective evaluations, including initialization and scouts. Algorithm 2 specifies the neighborhood and state updates within the framework of Algorithm 1. The procedure below specifies the modification to Algorithm 1. Figure 1 connects the gain–loss state calculation to branch selection, feasibility repair, and population updating; Table 1 summarizes the biological motivation and computational interpretation.
**Algorithm 2.** RSF-ABC neighborhood and state updateUse the initialization, phases, onlooker selection, scout rule, and stopping condition of Algorithm 1.For each employed or onlooker trial, read the current source’s stored gain *G*, loss *L*, and budget *B*.Compute *p*_explore_ = 1/[1 + exp(*κB*)] and draw a uniform random number *v* in [0,1].If *v* < *p*_explore_, apply the clipped Student-*t*_3_ exploratory coordinate move; otherwise apply the best-guided move with *ρ*∼𝒰(0,0.5).Repair the complete trial vector and evaluate its cost-aware Omega, turnover, gain, loss, and budget. Count this as one objective evaluation.Apply the common acceptance rule. On acceptance, store the trial’s weights and state; otherwise retain the incumbent’s weights and state.Update the trial counter and best-so-far source as in Algorithm 1. Recompute the state whenever a scout installs a new source.Return the best feasible portfolio when the common evaluation budget is exhausted.

Table 1 links each biological motivation to an explicit algorithmic choice and the comparison used to examine it. Its final column is an evaluation map for the ablations in Section 2.6 and the diagnostics in Section 3.2 and Section 3.3. The distribution, numerical settings, and repair operator are engineering choices; the biological analogy motivates the state-to-action relationship.

### 2.5. Computational Complexity

The problem combines discrete support selection with continuous weight allocation. There are NK candidate supports, so exhaustive support enumeration grows combinatorially when *K* varies with *N*. Cardinality-constrained portfolio models are known to create difficult mixed-integer search problems [9,10]. Continuous Omega reformulations [23] do not remove the binary selection decisions in this model. This motivates a fixed-budget heuristic, without asserting a polynomial-time global-optimality guarantee or a hardness proof specific to every Omega instance.

For the dense implementation, evaluating one candidate’s scenario returns costs *O*(*TN*); computing gains, losses, and turnover adds *O*(*T* + *N*). Selecting the top *K* coordinates by sorting costs *O*(*N*log*N*), and a bounded-simplex projection with *I* iterations costs *O*(*IK*). The state and sigmoid reuse the already computed gain and loss terms and add constant work. Including rank-based source selection, a conservative bound for a run is

*O* (*F*[*TN* + *N*log*N* + *IK* + *P*]). (12)

The return matrix, population, and batched scenario scores require *O*(*TN* + *PN* + *TP*) memory; stored diagnostic traces add up to *O*(*F*). These bounds concern one optimization run. A monthly backtest repeats that work over its holding windows and seeds. Fixed-budget timing and memory measurements in Section 3.5 complement this analysis; they do not establish that objective quality remains constant as dimension increases.

### 2.6. Ablations and Comparator Algorithms

Standard ABC is the portfolio implementation of the canonical neighborhood [3] described in Section 2.3. The other named ABC controls are ablations defined in this study, rather than independently published algorithms. HT-ABC always uses the clipped heavy-tailed move. RS-Light-ABC retains the budget-dependent switch but replaces heavy-tailed exploration with the canonical uniform multiplier. Fixed-Mix-ABC uses the same clipped heavy-tailed and conservative branches as RSF-ABC with a constant exploration probability of 0.4325421655. This probability is calibrated to the pooled realized heavy-branch frequency over 12 formation-only dates and 30 seeds, without holding-month returns.

The Fixed-Mix probability is a global matching device for comparing branch allocation. Because its diagnostic dates span the study period, it is not presented as a probability that would have been available prospectively at every earlier rebalance. Its role is to control average branch composition. All ABC variants share repair, objective, population size, scout limit, replacement rule, and evaluation budget. Table 2 brings their parameter settings together with those of the other optimizers.

Particle Swarm Optimization (PSO) [29] and Differential Evolution (DE) [30] use the same population size, initial feasible population, repair function, objective, and 3000-evaluation budget as the ABC variants. PSO decreases inertia linearly from 0.9 to 0.4 and uses cognitive and social coefficients of 1.49618. DE uses rand/1/bin mutation with differential weight 0.5 and crossover probability 0.9. Both methods use turnover to resolve numerical objective ties. These settings define the comparison; equal evaluation budgets do not establish that every optimizer is optimally tuned.

Four financial comparators address different practical alternatives. Equal weight over all *N* industries (EW-N) holds all industries at equal weight and is intentionally not cardinality-matched, providing the established naive-diversification reference [28]. Equal weight over *K* selected industries (EW-K) ranks industries by individual formation-window Omega, breaks ties by mean return, selects *K* industries, and assigns equal weights to the bounded support. MV-K solves a continuous long-only minimum-variance problem, selects its *K* largest weights, and re-solves minimum variance on that support. Omega-MILP solves the same empirical cost-aware Omega objective with binary selection and weight bounds using Dinkelbach iterations and the HiGHS mixed-integer solver. When the faster Omega-minus-one formulation indicates that the optimum may lie below one, an exact scenario-sign formulation represents positive and negative parts separately. Each monthly Omega-MILP call has a 30 s limit and a relative MIP-gap target of 0.001. Including these methods prevents a metaheuristic-only evaluation when Omega may admit a strong deterministic formulation [23].

### 2.7. Data and Monthly Walk-Forward Protocol

The experiments use daily value-weighted returns for the 10-, 30-, and 49-industry classifications from the Kenneth R. French Data Library [31]. The main universe contains 49 industry portfolios; the 10- and 30-industry data provide alternative levels of industry aggregation. They are separate published classifications, not simple subsets of 49 individual assets. The sample extends from 3 January 2006 to 31 December 2025 and contains 5031 complete trading-day observations, with returns expressed as decimals. These data represent industry portfolios rather than individual tradable stocks. The data library documents industry assignments and return construction, supporting reproducible selection of the input series.

Each rebalance uses the immediately preceding 504 trading days as its formation window and evaluates the selected portfolio over the next calendar month. There are 215 non-overlapping holding windows and 4508 out-of-sample trading days, from February 2008 through December 2025. Formation and holding indices do not overlap. Thresholds, covariance estimates, asset rankings, and target weights use the current formation data and pre-trade portfolio. The Fixed-Mix matching probability has the retrospective diagnostic role described in Section 2.6.

On the first day of a holding month, gross portfolio return *r_p_*_,1_ is converted to net return as

(13) $${\tilde{r}}_{p,1}=(1-\mathrm{cq})(1+r_{p,1})-1.$$

**Figure 2:**
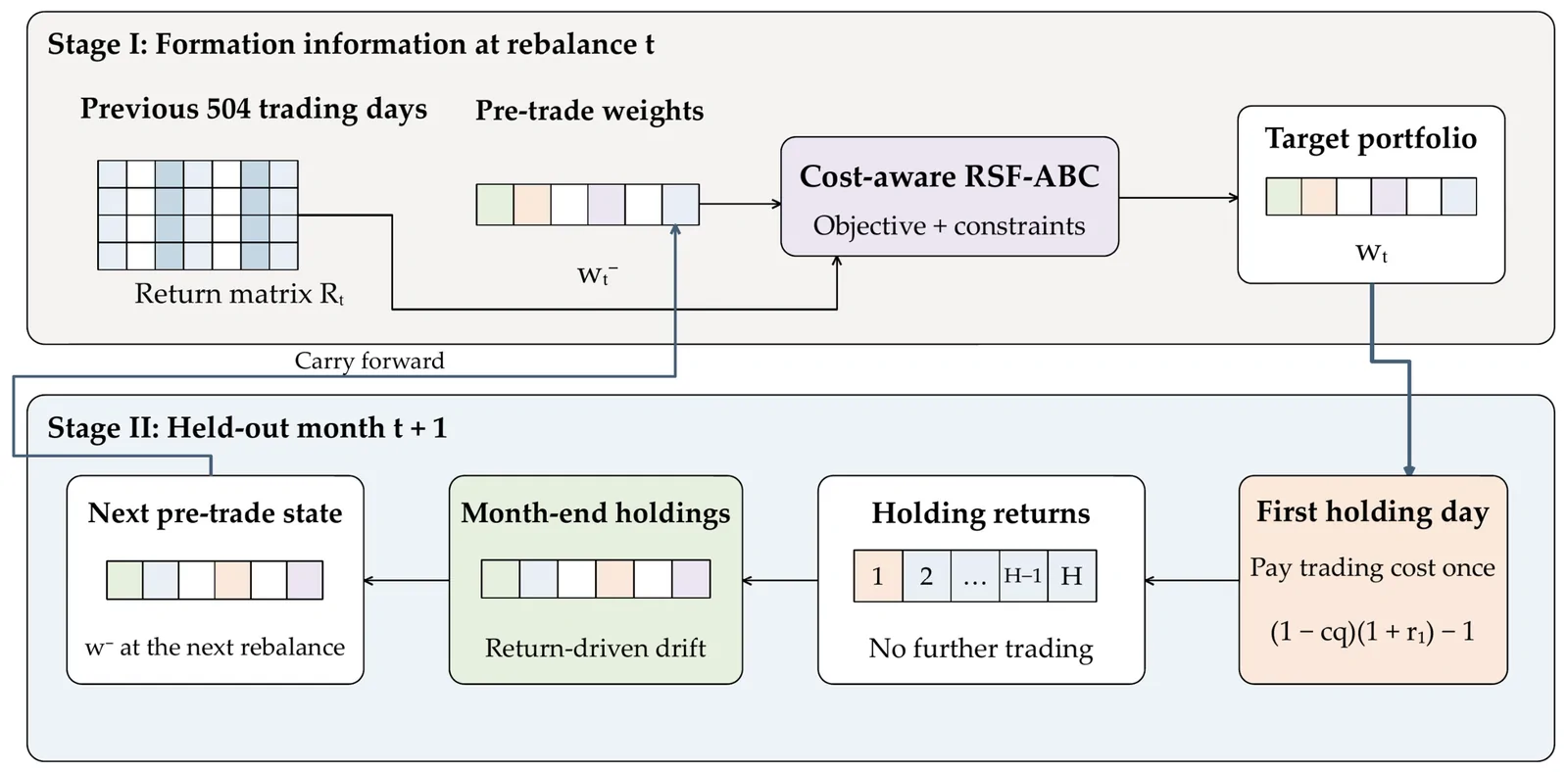
Monthly walk-forward evaluation and portfolio feedback. Stage I uses the preceding 504 trading days and pre-trade weights to select the target portfolio. Stage II applies the proportional transaction cost once on the first holding day and evaluates returns over the held-out month. Return-driven drift determines the pre-trade weights at the next rebalance. Formation and holding indices do not overlap. Black arrows show operations within each stage. Blue arrows pass the target weights to the holding month and carry the month-end portfolio forward to the next rebalance. Purple marks optimization, peach marks the first-day cost, and green marks month-end holdings. Gray and pale-blue backgrounds distinguish formation from evaluation.

Later daily returns in the same month are gross returns because no further trading is assumed. Realized end-of-month asset values determine the next pre-trade risky and cash weights. Turnover is therefore path-dependent: every target is compared with the portfolio actually carried into the rebalance, not with a fixed reference portfolio. Figure 2 summarizes the information boundary, one-time cost, holding period, and monthly feedback.

### 2.8. Experimental Design and Statistical Analysis

The main problem uses 49 industries, exactly 10 selected positions, active weights between 0.02 and 0.30, a 25 bp one-way cost, and a zero daily threshold. Five matched seeds (101, 202, 303, 404, and 505) are used for each of the seven stochastic methods. Four financial comparators provide equal-weight, minimum-variance, and mixed-integer alternatives. Seven boundary problems change one specification at a time: (*N*, *K*) = (10, 5), (30, 10), (49, 5), or (49, 15); a 10 bp or 50 bp one-way cost in the main universe; or a threshold equal to the formation-window mean of the equal-weight portfolio.

A separate computational diagnostic extends the universe to *N* = 100 and 200 through factor-preserving replication, with *K* = 20 and 30, respectively. These expanded cases test numerical scaling, rather than performance on independent market datasets. The population size is 30 and the objective-evaluation budget is 3000. Section 3.5 distinguishes the available runtime and memory summaries from the missing comparative measurements.

A formation-only diagnostic isolates search behavior from realized holding-month returns. It evaluates 12 evenly spaced rebalance dates, 30 seeds (1001–1030), matched initial populations, and the same 3000-evaluation budget for every stochastic method. The diagnostic contains 360 runs for each of RSF-ABC, Standard ABC, Fixed-Mix-ABC, HT-ABC, RS-Light-ABC, PSO, and DE. Holding-month returns do not enter the diagnostic or the Fixed-Mix calibration.

Formation-window summaries and paired-date effects answer different questions. Pooled run-level medians are reported descriptively and are not substituted for paired-date effects. The reported inferential comparisons concern the complete held-out return paths described below. Switching steepness is assessed separately within its two-comparison sensitivity family.

The primary financial outcome is complete-path net Omega at a zero daily threshold, calculated from all 4508 held-out returns. The primary effect is the mean matched-seed difference in this quantity over the five seeds, evaluated against Standard ABC and Fixed-Mix-ABC. Annualized return, volatility, zero-risk-free-rate Sharpe ratio, Sortino ratio, drawdown, daily CVaR, and turnover are secondary outcomes, defined in Section 2.9. Formation objective values are not treated as out-of-sample financial outcomes.

The paired bootstrap represents serial dependence and variation across algorithm seeds [32]. Each of the 10,000 bootstrap replicates samples five matched seed clusters with replacement. Within each sampled cluster, common 21-day moving-block indices are applied to the two methods’ return paths. Complete-path Omega is recomputed, paired seed differences are averaged, and the 2.5th and 97.5th percentiles form the 95% interval. The two primary comparisons use two-sided, plus-one-corrected bootstrap sign probabilities and constitute one Holm family. At the 5% familywise level, the smaller raw *p* value is compared with 0.025 and, if rejected, the larger with 0.05.

### 2.9. Definitions of Performance Measures

Let *r_d_* denote the net return on held-out day *d*, *D* = 4508, $\bar{r}$ the arithmetic mean daily return, and *s_r_* its sample standard deviation with denominator *D* − 1. There are *M* = 215 holding months. Annualization uses 252 trading days. Net Omega is the ratio of mean positive to mean negative net daily returns, with the same numerical stabilizer as the formation objective and a zero evaluation threshold.

Annualized return is geometric, whereas the Sharpe numerator uses the annualized arithmetic mean:

(14) $$R_{\mathrm{ann}}={\left[\prod_{d=1}^{D}(1+r_{d})\right]}^{252/D}-1.$$

(15) $$\sigma_{\mathrm{ann}}=\sqrt{252}\;s_{r},\;\;S_{0}=\frac{252\bar{r}}{\sigma_{\mathrm{ann}}}.$$

Thus, volatility is the annualized standard deviation of net daily returns. “Zero-RF Sharpe” means that the risk-free return is set to zero; it is not a Sharpe ratio computed using a Treasury-bill series. Sortino uses a zero target and the root mean square of daily shortfalls:

(16) $${\mathrm{Sortino}}_{0}=\frac{\sqrt{252}\;\bar{r}}{\sqrt{D^{-1}\sum_{d=1}^{D}\min(r_{d},0{)}^{2}}}.$$

For cumulative wealth $V_{d}=\prod_{h=1}^{d}(1+r_{h})$ with *V*_0_ = 1, maximum drawdown is

(17) $$\mathrm{MDD}=\underset{1\leq d\leq D}{\min}\left\{\frac{V_{d}}{\underset{0\leq h\leq d}{\max}V_{h}}-1\right\}.$$

It is reported as a negative return, so a value closer to zero is preferable. Daily CVaR95 is the empirical mean of daily losses −*r_d_* at or above their empirical 95th percentile. Monthly turnover is the arithmetic mean of the *M* one-way turnover values defined in the model. Returns, volatility, drawdown, and CVaR are reported as decimals; Omega, Sharpe, and Sortino are unitless. The geometric return should not be substituted into the Sharpe formula.

Feasibility rate is the fraction of evaluated output portfolios satisfying their assigned investment, cardinality, and weight-bound constraints. Runtime is wall-clock optimization time per rebalance, reported in seconds; peak resident memory is reported in MB. The unconstrained EW-N diversification reference is interpreted under its own all-industry specification.

### 2.10. Implementation and Reproducibility

The reference implementation uses Python 3.12 with NumPy 2.5.1, pandas 3.0.5, SciPy 1.18.0, and joblib 1.5.3; SciPy’s HiGHS interface solves the mixed-integer comparator. The experiments use a custom-built desktop workstation with an AMD Ryzen 9 5900X processor, 12 physical cores, two hardware threads per core (24 logical threads), a 3.7 GHz base frequency, and 64 GB RAM. The reported execution setup uses six isolated CPU workers, one numerical-library thread per process, and no GPU. Independent runs are scheduled across workers; each neighborhood search remains sequential. The public implementation includes checks for constraints, objectives, dates, data parsing, branch selection, repair, and walk-forward accounting. Section 3.5 reports the available computational summaries.

## 3. Results

### 3.1. Out-of-Sample Performance and Trading Profile

The main outcome set contains 35 stochastic paths and four deterministic paths, each spanning 215 holding windows and 4508 out-of-sample trading days. The constrained portfolios satisfy full investment, exact cardinality, and active-weight bounds. EW-N is the explicitly unmatched all-industry diversification reference. Table 3 separates performance measures from risk and trading measures for readability. Stochastic entries are means over five complete paths; each deterministic comparator contributes one path.

**Figure 3:**
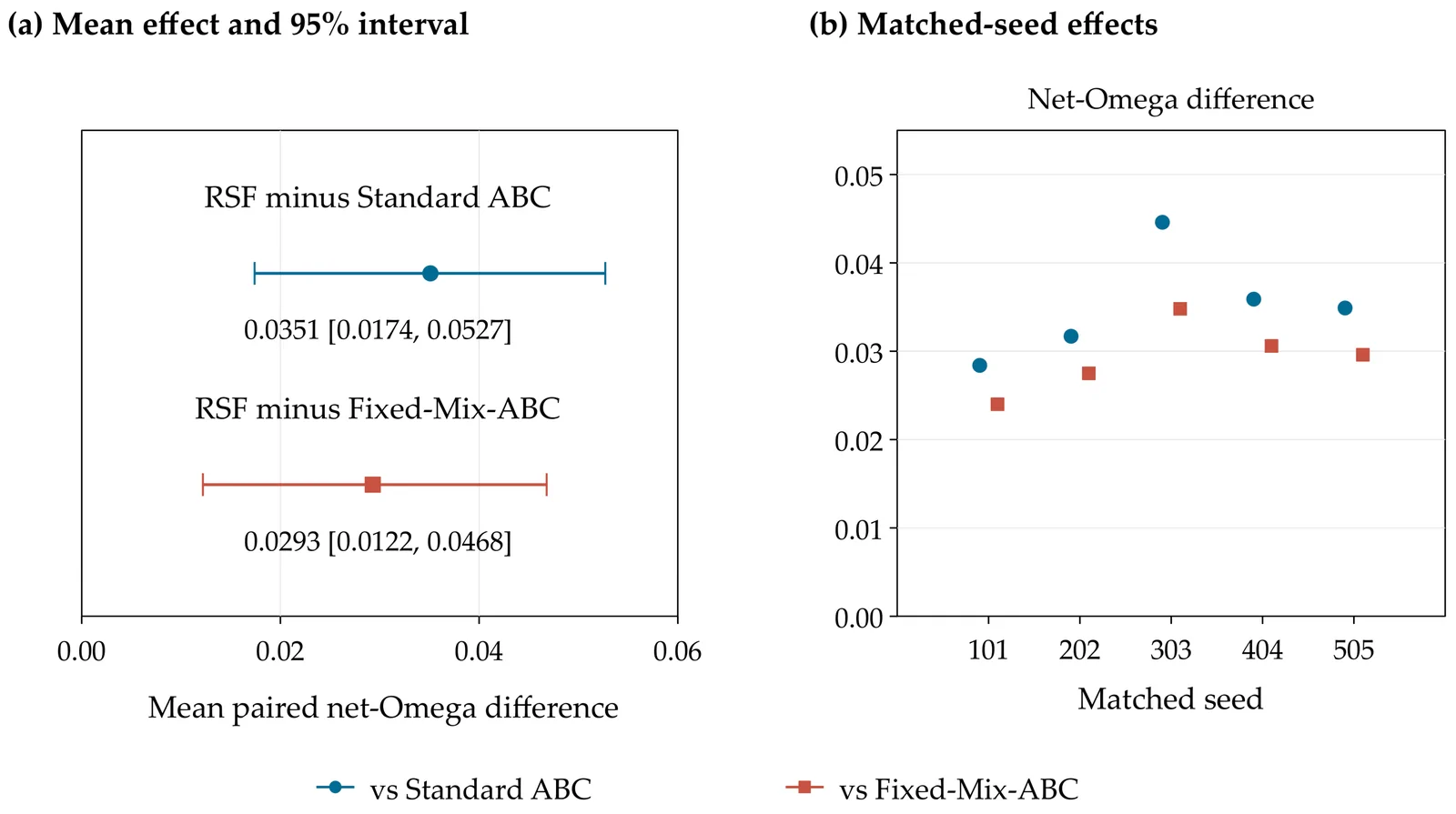
Paired comparisons for 49 industries, *K* = 10, and a 25 bp one-way cost. (**a**) Mean complete-path net-Omega differences with 95% hierarchical paired-bootstrap intervals shown as horizontal bars. (**b**) Differences for the five matched seeds. In both panels, blue circles denote RSF-ABC minus Standard ABC, and red squares denote RSF-ABC minus Fixed-Mix-ABC. Positive values favor RSF-ABC. Raw *p* values and the two-comparison Holm rule are reported in the text.

RSF-ABC has the highest mean complete-path net Omega: 1.1635, compared with 1.1284 for Standard ABC and 1.1342 for Fixed-Mix-ABC. The matched-seed differences are 0.0351 against Standard ABC (95% interval 0.0174–0.0527; raw two-sided *p* = 0.004) and 0.0293 against Fixed-Mix-ABC (95% interval 0.0122–0.0468; raw *p* = 0.006). Both raw *p* values pass the two-comparison Holm procedure specified in Section 2.8 at the 5% familywise level. Figure 3 displays mean effects, interval estimates, and the five matched-seed differences.

The five RSF-ABC-minus-Standard-ABC differences are 0.0284, 0.0317, 0.0446, 0.0359, and 0.0349; the corresponding Fixed-Mix differences are 0.0240, 0.0275, 0.0348, 0.0306, and 0.0296. All are positive. Their common direction supports the main comparison across the five seeds, while the small number of complete paths limits precision about algorithmic variability.

Outside the ABC family, Table 3 gives net Omega of 1.1410 for PSO, 1.1450 for DE, and 1.1510 for Omega-MILP. The corresponding RSF-ABC differences are 0.0225 (1.97%), 0.0185 (1.62%), and 0.0125 (1.09%), where relative differences use each comparator as the denominator.

The secondary outcomes point in the same direction without making RSF-ABC dominant on every risk dimension. RSF-ABC reached an annualized return of 0.1362, annualized volatility of 0.1910, a zero-risk-free-rate Sharpe ratio of 0.7430, a Sortino ratio of 1.0870, maximum drawdown of −0.3560, and daily CVaR95 loss of 0.0287. Minimum-variance selection retained lower volatility and CVaR, so the proposed method’s main advantage is the gain–loss trade-off measured by Omega rather than universal risk minimization.

RSF-ABC has mean monthly one-way turnover of 0.4780, compared with 0.5570 for Standard ABC and 0.5020 for Fixed-Mix-ABC. This is not the lowest turnover in Table 3: PSO, DE, and Omega-MILP have values of 0.2560, 0.3150, and 0.1280. Thus, the reported net-Omega improvement should be interpreted alongside the additional trading relative to these alternatives.

### 3.2. Formation-Window Diagnostics

The formation-only diagnostic separates search behavior from realized investment returns. Across 360 runs per method, median terminal formation Omega was 1.3948 for RSF-ABC, 1.3471 for Fixed-Mix-ABC, and 1.2640 for Standard ABC. The corresponding means were 1.3821, 1.3384, and 1.2588. These summaries characterize fixed-budget optimization.

**Figure 4:**
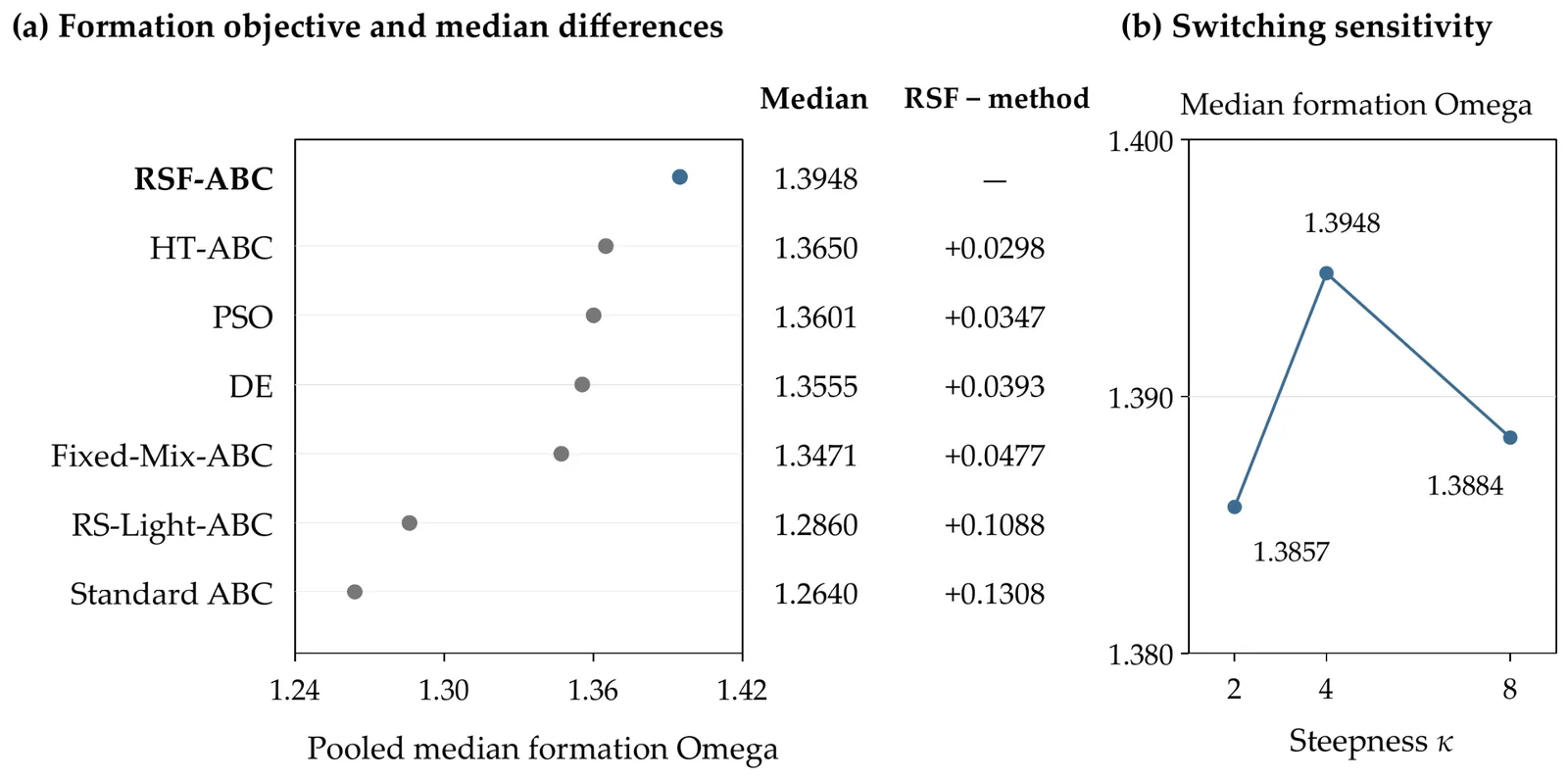
Reported formation objective and switching sensitivity. (**a**) Pooled median terminal formation Omega for seven stochastic methods, with the numerical medians and descriptive differences relative to RSF-ABC aligned by method. (**b**) Median formation Omega at *κ* = 2, 4, and 8. Differences in pooled medians are descriptive contrasts, not paired-date inference. In panel (**a**), the blue dot identifies RSF-ABC and gray dots identify the six comparison methods. In panel (**b**), blue dots show the three RSF-ABC sensitivity settings, the blue line connects these settings, and the offset numerical labels report their medians.

The pooled median difference between RSF-ABC and Fixed-Mix-ABC is 0.0477 (1.3948 minus 1.3471). The corresponding differences against HT-ABC and RS-Light-ABC are 0.0298 and 0.1088. Figure 4 reports these descriptive differences explicitly as differences in pooled medians. They summarize the run-level distributions.

The reported controller summaries are consistent with the specified response rule. RSF-ABC has mean normalized budget 0.0690, mean exploration probability 0.4321, and realized heavy-branch frequency 0.4326; the mean within-run budget–probability correlation is −0.99995. Fixed-Mix-ABC uses probability 0.43254 and has realized frequency 0.4331. Agreement between probabilities and frequencies checks activation of the controller.

The three ablations address complementary explanations. Comparing RSF-ABC with RS-Light-ABC examines the heavy-tailed kernel inside the switching framework; HT-ABC removes the conservative branch; and Fixed-Mix-ABC removes state dependence while retaining both branches. RSF-ABC has the higher pooled median in each comparison. The out-of-sample Fixed-Mix comparison in Section 3.1 provides the separate evidence that the state-conditioned allocation is useful for the portfolio paths.

### 3.3. Sensitivity to Switching Steepness

The sensitivity analysis varies κ over 2, 4, and 8 while retaining the remaining design. These values test a shallower response, the primary response, and a steeper response around the same zero-budget midpoint. The median terminal formation Omega values are 1.3857, 1.3948, and 1.3884, respectively. Relative to κ = 2 and κ = 8, the primary setting has gains of 0.0091 and 0.0064; the corresponding Holm-adjusted paired-test *p* values are 0.031 and 0.044 for this two-comparison sensitivity family. Figure 4b displays the three objective summaries. The result favors κ = 4 within this local diagnostic, but does not establish a universally optimal slope.

### 3.4. Robustness and Boundary Conditions

**Figure 5:**
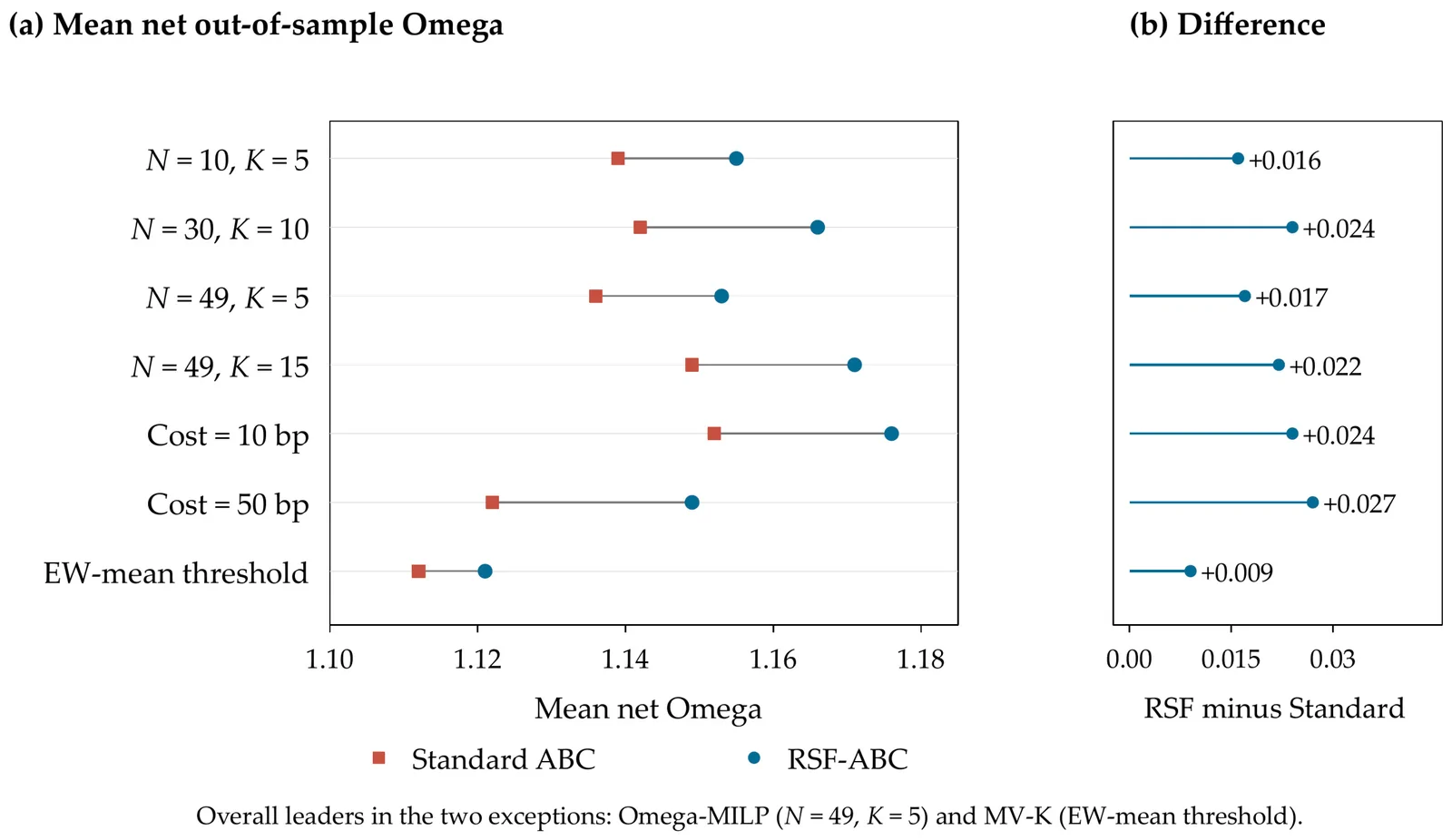
Seven boundary problems. (**a**) Mean net out-of-sample Omega for RSF-ABC and Standard ABC. (**b**) Their signed differences. All settings retain the main specification except the condition shown. RSF-ABC exceeds Standard ABC in all seven comparisons. The best method is RSF-ABC in five conditions, Omega-MILP at *N* = 49 and *K* = 5, and MV-K under the equal-weight-mean threshold. Blue circles represent RSF-ABC and red squares represent Standard ABC. Gray horizontal segments in panel (**a**) connect the two results for the same condition; blue horizontal segments in panel (**b**) extend from zero to the signed RSF-ABC-minus-Standard-ABC difference.

RSF-ABC exceeded Standard ABC in all seven boundary conditions. Differences ranged from 0.009 under the formation-window equal-weight-mean threshold to 0.027 under 50 bp costs. RSF-ABC was the highest-Omega method in five conditions; deterministic comparators were highest in the *N* = 49, *K* = 5 condition and under the alternative threshold. The result supports a broad operating range without implying that one metaheuristic dominates every formulation. Figure 5 compares RSF-ABC with Standard ABC across these seven conditions.

The deterministic comparators remain important. Omega-MILP produced the highest Omega in one cardinality condition, and minimum-variance selection led under the alternative threshold. These outcomes show why feasibility across the tested dimensions must be assessed alongside simple, classical, and deterministic financial baselines.

### 3.5. Computational Cost and Scaling

**Figure 6:**
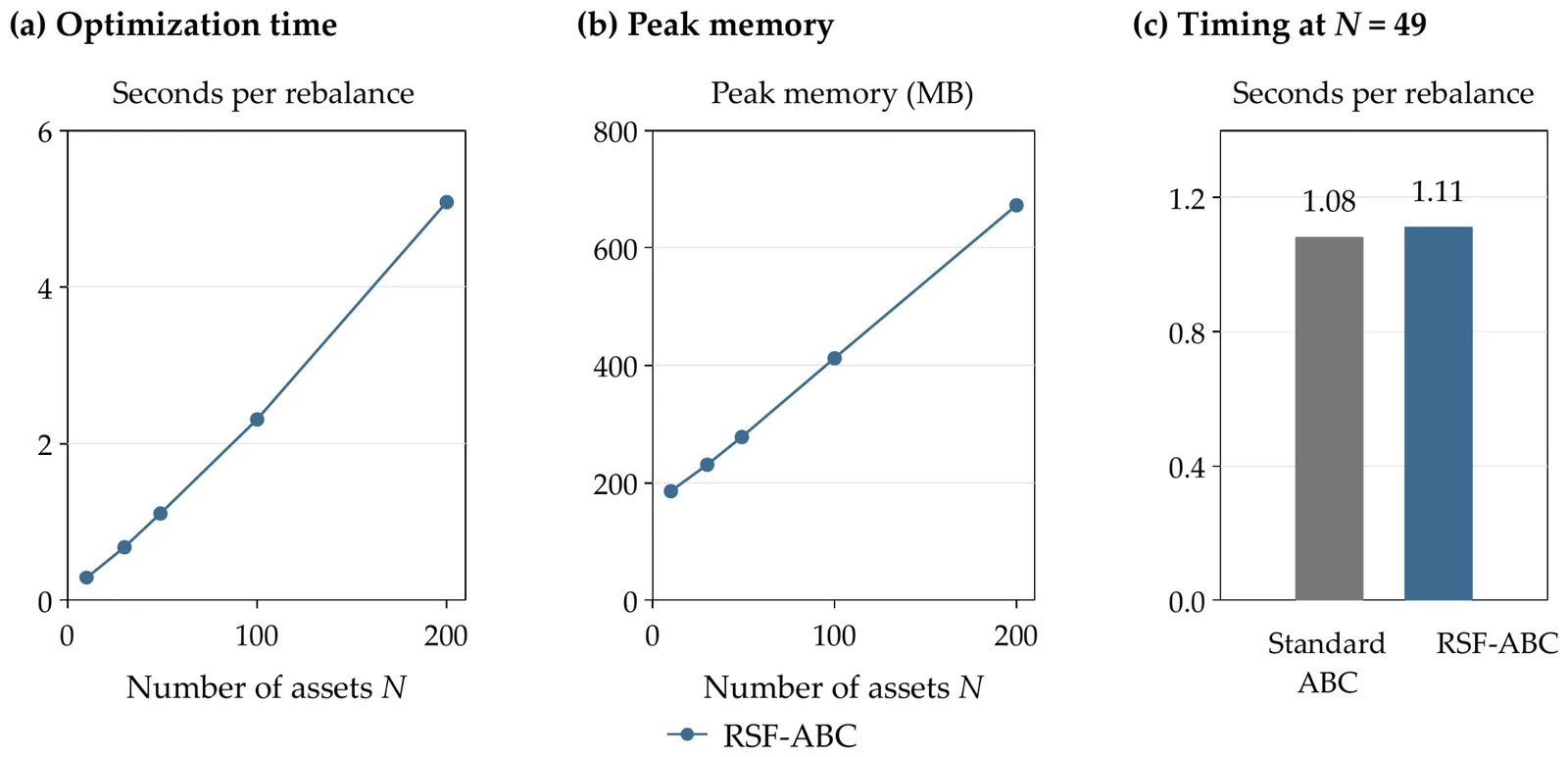
Available computational summaries at a fixed budget of 3000 evaluations. (**a**) RSF-ABC median time per rebalance. (**b**) RSF-ABC reported peak memory. (**c**) Median times for Standard ABC and RSF-ABC at *N* = 49. Blue marks indicate RSF-ABC, the gray bar in panel (**c**) indicates Standard ABC.

Figure 6 reports the available computational summaries under a fixed budget of 3000 objective evaluations. For *N* = 10, 30, 49, 100, and 200, RSF-ABC median optimization times are 0.29, 0.68, 1.11, 2.31, and 5.08 s per rebalance, and reported peak-memory summaries are 186, 231, 278, 412, and 672 MB. The only corresponding Standard ABC summary is its 1.08 s median at *N* = 49. Thus, RSF-ABC takes 0.03 s, or 2.8%, longer at that dimension. The larger universes use replicated factors, and the reported feasibility rate is 100%.

## 4. Discussion

### 4.1. Interpretation of the State-Conditioned Comparison

The principal methodological result concerns feedback within a specified search design. The matched-mixture control uses the same heavy-tailed and best-guided branches, repair operator, objective, and evaluation budget. Its lower net Omega therefore supports the value of the evaluated state-dependent allocation rule within this implementation. Table 3 also includes particle swarm optimization, differential evolution, and a mixed-integer Omega comparator. Their presence extends the evaluation beyond bee-colony variants, while the modest descriptive margins and incomplete resource comparison limit the scope of the evidence.

The five paired effects have the same positive sign, and both 95% intervals exclude zero. The paired bootstrap aligns uncertainty with complete-path Omega and preserves matching between methods.

Controller activation and controller value are distinct observations. The negative budget–probability correlation follows closely from the specified sigmoid and verifies that the implementation responds to its input. The formation summaries and the held-out Fixed-Mix comparison address those outcomes separately.

### 4.2. Relation Between the Objective and Search State

The budget is constructed from the same gain and loss terms as Omega. Ignoring the very small stabilizer, we can write it as *B* = (Ω − 1)/(Ω + 1). It therefore rescales the candidate’s current gain–loss ratio rather than introducing an independent market predictor. Its algorithmic role is to make that balance a bounded input to the branch-selection rule. An adverse balance raises the frequency of heavy-tailed proposals, while a favorable balance increases best-guided search.

The control design helps identify which implementation change accompanies the improvement. Standard ABC changes both the kernel and its allocation relative to RSF-ABC, so that comparison alone cannot isolate state conditioning. RS-Light-ABC, HT-ABC, and Fixed-Mix-ABC narrow the comparison progressively. The pooled formation summaries remain descriptive; the primary financial inference concerns the complete-path comparison with the two prespecified controls.

Formation quality and held-out financial performance remain distinct, even when both favor the same method. Rolling estimates can be noisy, and the next month may follow a different distribution. The study therefore retains the full walk-forward path, portfolio drift, one-time trading cost, and matched-seed inference rather than treating a higher formation objective as sufficient evidence.

### 4.3. Feasibility, Trading, and Scaling

The exact-cardinality repair retains feasibility across the tested dimensions. At 49 assets, the proposed controller takes 1.11 s per rebalance compared with 1.08 s for Standard ABC. This is an additional 0.03 s (2.8%), not a speed improvement. The higher net Omega obtained at a shared evaluation budget must therefore be interpreted together with this added runtime. Wall-clock time alone does not determine CPU utilization or memory consumption.

The available RSF-ABC profile increases from 0.29 s and 186 MB at 10 assets to 5.08 s and 672 MB at 200 assets. The larger cases use factor-preserving replication, so this result concerns array size rather than financial generalization. Missing Standard ABC scaling and memory profiles prevent a comparative resource-efficiency conclusion. Equal objective-evaluation counts also need not imply equal computational effort, a distinction emphasized in optimization-benchmark methodology [33].

Repair can change the relationship between a coordinate-level proposal and the final portfolio support. The budget is computed before the action, while repair determines the feasible support afterward. Future work could examine support-aware proposals or states incorporating prospective turnover, and compare them under the same evaluation and tuning budgets.

### 4.4. Financial Scope and Limitations

The experiment is based on U.S. industry portfolios and a historical monthly protocol. Its findings do not by themselves establish performance for individual stocks, international assets, higher-frequency trading, or live execution. A constant proportional charge is a useful controlled approximation, but market impact can change the relationship between historical evaluation and deployed trading, as emphasized in robust reinforcement-learning studies [17].

The differences outside the ABC family are modest. In Table 3, RSF-ABC exceeds PSO, DE, and Omega-MILP in net Omega by 0.0225, 0.0185, and 0.0125, respectively. These are descriptive differences: the reported primary inferential family covers Standard ABC and Fixed-Mix-ABC only. MV-K retains lower volatility and daily CVaR, and deterministic comparators lead in two boundary conditions. The results therefore motivate further evaluation of state conditioning under the specified objective, rather than a claim of broadly superior portfolio optimization.

Omega is threshold-dependent [22,25,26,27]. The alternative threshold broadens the evaluation but does not cover distributional ambiguity or all economically relevant targets. Likewise, the κ diagnostic tests local sensitivity, rather than joint parameter robustness. The Fixed-Mix probability is calibrated retrospectively for mechanism analysis; a deployable comparison requires calibration confined to preceding data. These limitations motivate the prospective tests in Section 4.5.

### 4.5. Broader Research Connections and Future Work

A broader unresolved question is how an optimizer should use feedback when the financial objective is estimated from changing, noisy data. Recent work studies learned allocation policies [16,20,21], robustness to prediction errors [19], and uncertain trading impact [17]. The present gain–loss controller addresses a complementary level: it changes search behavior within a rebalance, without learning a market forecast. Whether this simple feedback remains useful when estimates shift, execution costs change, or another optimizer is used remains open.

The first next step is a prospective walk-forward comparison on independent stock and international-asset universes. At each date, calibrate the fixed-mixture probability and all tuning choices using preceding data only, then freeze them for the next holding month. Compare the gain–loss state with turnover-aware and support-aware alternatives under shared inputs and tuning budgets. Report paired net-Omega differences, turnover, and uncertainty separately across market regimes; this would test whether the controller’s contribution persists beyond retrospective branch-frequency matching.

The second step is to compare objective quality at matched wall-clock limits for bee-colony, particle-swarm, differential-evolution, and mixed-integer optimizers, recording peak resident memory and complete convergence traces [33]. Alongside equal-evaluation tests, this would show whether improved allocation quality offsets the controller’s overhead. The third step is to introduce spread and market-impact costs and uncertain return scenarios [17,27], then test whether improvements survive more demanding execution and estimation assumptions. These experiments address computational value, transferability, and economic usefulness as separate questions.

## 5. Conclusions

Risk-Sensitive Foraging Artificial Bee Colony uses the Omega objective’s normalized gain–loss balance to choose between heavy-tailed exploration and best-guided search. In the reported portfolio comparison, mean net Omega is 1.1635, versus 1.1284 for standard artificial bee colony and 1.1342 for a fixed-probability mixture of the same search moves. Particle swarm optimization and differential evolution yield 1.1410 and 1.1450, respectively. The proposed method leads in five of seven boundary conditions, while deterministic alternatives lead in two. The matched-mixture comparison supports the evaluated state-dependent control rule; the broader comparisons show modest gains under the stated objective.

These gains incur additional computation: at 49 assets, median optimization time is 1.11 s rather than 1.08 s for the standard bee-colony method. Complete comparative memory and scaling profiles remain necessary to assess resource efficiency. The evidence is limited to industry portfolios, proportional trading costs, five main seeds, local parameter sensitivity, and replicated-factor scaling cases. Prospective calibration, independent asset universes, matched-time comparisons, and execution-cost stress tests are the next steps for determining the controller’s practical value.

## Figures and Tables

**Table 1:** Biological motivation, computational implementation, and corresponding evaluation.

Biological Motivation	Computational Implementation	Evaluation
State-dependent responses to reward variability [1,2]	Budget *B* from the candidate’s Omega gain and loss terms	Compare RSF-ABC with the identical state-independent branch mixture
Smooth state response (engineering choice)	Sigmoid exploration probability; primary κ = 4	Record predicted and realized branch frequencies; vary κ over 2, 4, and 8
Variable actions under an adverse state	Clipped Student-t_3_ exploratory multiplier in [−4, 4]	Compare with HT-ABC and light-tailed switching
Less variable actions under a favorable state	Best-guided coordinate step with ρ in [0, 0.5]	Retain the same branch in Fixed-Mix-ABC
State-contingent action selection	Shared ABC phases, repair, objective, and budget	Separate controller activation, formation objective, and held-out portfolio outcomes

**Table 2:** Experimental conditions and optimizer parameters. Unless a row specifies a boundary condition or method, its setting is shared.

Item	Main or Shared Setting	Alternative or Interpretation
Data	49 value-weighted daily industry portfolios	10- and 30-industry classifications
Sample	3 January 2006 to 31 December 2025; 5031 complete days	Returns expressed as decimals
Evaluation	504-day formation; next calendar month held out	215 windows; 4508 held-out days
Constraints	Long-only; full investment; *K* = 10; active weights 0.02–0.30	(*N*, *K*) = (10, 5), (30, 10), (49, 5), (49, 15)
Trading cost	25 bp one way; charged once at rebalancing	10 and 50 bp; formation proxy uses H = 21
Omega	Daily threshold τ = 0; ε = 10^−12^	Alternative τ: formation equal-weight mean
Stochastic budget	Population P = 30; F = 3000 evaluations	Includes initialization and scouts
Numerical acceptance	Omega tie tolerance 10^−12^; lower turnover breaks ties	Same comparison rule for stochastic methods
ABC scaffold	Scout limit 100; descending-rank onlookers	Common greedy acceptance and repair
Standard ABC	Uniform multiplier in [−1, 1]	Basic ABC neighborhood
RSF-ABC	κ = 4; Student t with 3 degrees of freedom; clipping [−4, 4]	Conservative coefficient uniform in [0, 0.5]
HT-ABC	Always use the same clipped Student-t move	No conservative branch
RS-Light-ABC	Same κ = 4 switch and conservative branch	Exploration multiplier uniform in [−1, 1]
Fixed-Mix-ABC	Constant exploration probability 0.4325421655	Same two branches as RSF-ABC; global formation-only calibration
PSO	Inertia linearly 0.9 to 0.4; cognitive and social coefficients 1.49618	Same repair, objective, and evaluation budget
DE	rand/1/bin; differential weight 0.5; crossover probability 0.9	Same repair, objective, and evaluation budget
Omega-MILP	30 s monthly limit; relative MIP-gap target 0.001	Deterministic comparator; no equal evaluation-count claim
Main seeds	101, 202, 303, 404, 505	Five matched paths per stochastic method
Formation diagnostic	12 dates; seeds 1001–1030; seven stochastic methods	360 runs per method; matched initial populations
Switch sensitivity	κ = 2, 4, 8	Two comparisons against κ = 4
Primary inference	10,000 paired bootstrap replicates; 21-day blocks; five seed clusters	Two primary comparisons; Holm family
Scaling diagnostic	*N* = 10, 30, 49, 100, 200	Replicated-factor extensions: *K* = 20 and 30 at *N* = 100 and 200

**Table 3:** Main-condition results for the 49-industry universe with *K* = 10 and a 25 bp one-way cost. Panels (**a**) return and risk-adjusted performance and (**b**) risk and trading activity use the same method order. Stochastic entries are five-path means; deterministic entries represent one path. Arrows indicate the preferred direction. Definitions and units are given in Section 2.9.

(**a**)				
**Method**	**Net Omega ↑**	**Ann. Return ↑**	**Zero-RF Sharpe ↑**	**Sortino ↑**
RSF-ABC	1.1635	0.1362	0.7430	1.0870
Fixed-Mix-ABC	1.1342	0.1180	0.6530	0.9300
Standard ABC	1.1284	0.1152	0.6250	0.8860
HT-ABC	1.1420	0.1210	0.6650	0.9490
RS-Light-ABC	1.1320	0.1160	0.6310	0.8950
PSO	1.1410	0.1220	0.6800	0.9670
DE	1.1450	0.1240	0.6940	0.9900
EW-N	1.1201	0.1079	0.5926	0.8297
EW-K	1.1240	0.1135	0.6103	0.8456
MV-K	1.1317	0.0882	0.6385	0.8920
Omega-MILP	1.1510	0.1280	0.7240	1.0200
(**b**)				
**Method**	**Ann. Volatility ↓**	**Max Drawdown ↑**	**Daily CVaR95 ↓**	**Monthly Turnover ↓**
RSF-ABC	0.1910	−0.3560	0.0287	0.4780
Fixed-Mix-ABC	0.1965	−0.4120	0.0303	0.5020
Standard ABC	0.2022	−0.4310	0.0313	0.5570
HT-ABC	0.1970	−0.4010	0.0300	0.5220
RS-Light-ABC	0.2010	−0.4280	0.0310	0.5680
PSO	0.1950	−0.3980	0.0297	0.2560
DE	0.1940	−0.3910	0.0294	0.3150
EW-N	0.2104	−0.5481	0.0324	0.0216
EW-K	0.2137	−0.5748	0.0336	0.1673
MV-K	0.1501	−0.3676	0.0225	0.0986
Omega-MILP	0.1900	−0.3760	0.0289	0.1280

## Data Availability

Publicly available datasets were analyzed in this study. The daily value-weighted 10-, 30-, and 49-industry portfolio datasets used in this study are available from the Kenneth R. French Data Library at https://mba.tuck.dartmouth.edu/pages/faculty/ken.french/data_library.html (accessed on 10 July 2026). The source code, frozen experiment configurations, automated tests, and reproduction instructions are openly available at https://github.com/bubblewy/rsf-abc-portfolio-optimization (accessed on 10 July 2026). The repository scripts download the source data directly from the original provider; therefore, the third-party raw datasets are not redistributed in the repository and remain subject to the provider’s terms.

## Abbreviations

The following abbreviations and symbols are used in this manuscript:

**Abbreviation**	**Definition**
ABC	Artificial Bee Colony
CVaR	Conditional Value at Risk
DE	Differential Evolution
EW-K	Equal Weight over *K* Selected Industries
EW-N	Equal Weight over All N Industries
Fixed-Mix-ABC	State-Independent Matched-Mixture Artificial Bee Colony
HT-ABC	Heavy-Tail-Only Artificial Bee Colony
MILP	Mixed-Integer Linear Program
MV-K	Cardinality-Constrained Minimum Variance
Omega-MILP	Mixed-Integer Maximum-Omega Comparator
PSO	Particle Swarm Optimization
RSF-ABC	Risk-Sensitive Foraging Artificial Bee Colony
RS-Light-ABC	Risk-Sensitive Switching with Light-Tailed Exploration
**Nomenclature**	
Symbol	Definition
*N*, *K*	Number of available assets and required number of selected assets
*T*, *H*	Formation-window length and cost-amortization horizon, in trading days
*r_t_*, *w*	Daily asset-return vector and target portfolio-weight vector
*z_j_*, *ℓ*, *u*	Selection indicator and lower and upper active-weight bounds
𝒲	Set of fully invested, feasible target portfolios
*w*^−^, *a*^−^	Risky-asset and cash weights immediately before trading
*q*, *c*	One-way turnover and proportional one-way transaction-cost rate
*τ*, *x_t_*	Daily Omega threshold and cost-adjusted excess return
*G*, *L*, Ω	Mean threshold gain, mean threshold loss, and Omega ratio
*ε*	Positive numerical stabilizer in ratio denominators
*B*, *κ*, *p*_explore_	Normalized gain–loss state, sigmoid steepness, and exploration probability
*P*, *F*, *I*	Population size, objective-evaluation budget, and projection iterations
*i*, *k*, *j*	Source, partner, and selected coordinate indices; k ≠ i
*w*^⋆^, *ϕ*, *ρ*	Best source, exploratory multiplier, and best-guided step coefficient
*D*, *M*, *r_d_*, *V_d_*	Held-out days, holding months, net daily return, and cumulative wealth
